# Cerebrospinal fluid irisin and its correlation to Alzheimer's disease biomarkers in a Brazilian dementia cohort

**DOI:** 10.1002/alz70856_107065

**Published:** 2026-01-08

**Authors:** Alice da Luz Saldanha, Guilherme B. de Freitas, Felipe K. Sudo, Carlos O Brandão, Bart Vanderborght, Mychael V. Lourenco, Fernanda Tovar‐Moll, Paulo E. Mattos, Luis E. Santos, Fernanda Guarino De Felice

**Affiliations:** ^1^ D'Or Institute for Research and Education, Rio de Janeiro, Rio de Janeiro, Brazil; ^2^ Federal University of Rio de Janeiro, Rio de Janeiro, Brazil; ^3^ Queen's University, Kingston, ON, Canada; ^4^ D'Or Institute for Research and Education, Rio de Janeiro, RJ, Brazil; ^5^ D'Or Institute for Research and Education (IDOR), Rio de Janeiro, RJ, Brazil; ^6^ Neurolife, Rio de Janeiro, RJ, Brazil; ^7^ Institute of Medical Biochemistry Leopoldo de Meis, Federal University of Rio de Janeiro, Rio De Janeiro, Rio de Janeiro, Brazil; ^8^ Federal University of Rio de Janeiro, Rio de Janeiro, RJ, Brazil

## Abstract

**Background:**

Physical exercise (PE) is pointed as a potential nonpharmacological preventative and interventional strategy to slow down the decline of cognition, both in clinically healthy individuals and patients with Alzheimer's disease (AD). Irisin, produced by skeletal muscle during PE has shown to play a role in the nervous system, and it is speculated that it may have a neuroprotective role.

**Methods:**

Using a well‐established Brazilian cohort, we selected samples from 25 cognitively unimpaired and 27 cognitively impaired older adults (aged 55+) ‐ diagnosed with amnestic mild cognitive impairment (aMCI) or Alzheimer's disease. In addition to clinical diagnosis, amyloid status was used to stratify participants, using CSF biomarker cutoffs established for the cohort. Using the Single Molecule Array (SIMOA) platform, NfL, GFAP, IL‐6 were measured in plasma and pTau181 and pTau217 in CSF. Statistical analysis was performed using GraphPad Prism 9 and Rstudio.

**Results:**

A significant correlation was observed between CSF irisin, pTau181 and pTau217 levels in control individuals, with those with the highest levels of pTau also having the highest irisin. Intriguingly, this effect was not present in the cognitively impaired group. In our cohort, correlation data between CSF irisin and inflammatory plasma biomarkers did not show significance, except for IL‐6 in the aMCI group and NfL in the AD group.

**Conclusion:**

In conclusion, our results may suggest that high levels of irisin could protect participants with abnormal CSF pTau from cognitive impairment. These data point to irisin as a potential resilience biomarker in amyloid positive older individuals.

